# Preservative Effectiveness of Lactic Acid Bacteria on Fruits and Vegetables

**DOI:** 10.1155/ijcb/5833236

**Published:** 2025-07-31

**Authors:** Desalegn Amenu, Ayantu Nugusa, Temesgen Tafesse

**Affiliations:** ^1^College of Natural and Computational Sciences, Biology Department, Wollega University, Nekemte City, Oromia, Ethiopia; ^2^Ministry of Health, Armauer Hansen Research Institute, Addis Ababa, Ethiopia

**Keywords:** bacteriocins, biotechnology, exopolysaccharides, fermentation, food safety, fruits, lactic acid bacteria, preservation, shelf life, vegetables

## Abstract

**Background:** Lactic acid bacteria (LAB) are extensively used in the preservation of fruits and vegetables due to their ability to inhibit spoilage organisms and pathogenic bacteria. The fermentation process mediated by LAB not only extends the shelf life of produce but also enhances its nutritional and sensory properties.

**Objective:** This study is aimed at reviewing the mechanisms through which LAB preserve fruits and vegetables, evaluating the benefits of their application, and highlighting recent advances in this field.

**Methods:** A comprehensive literature review was conducted, focusing on the various preservative mechanisms of LAB, including acid production, bacteriocin secretion, competition for nutrients, and exopolysaccharide production. The impact of LAB on the shelf life, safety, nutritional value, and sensory attributes of fruits and vegetables was assessed through an analysis of recent scientific studies and case examples.

**Results:** LAB extends the shelf life of fruits and vegetables primarily through the production of lactic acid, which lowers the pH and inhibits the growth of spoilage organisms and pathogens. The secretion of bacteriocins by LAB provides additional antimicrobial activity. Competition for nutrients further suppresses unwanted microbial growth. Exopolysaccharides produced by LAB can improve texture and form protective biofilms. Notable examples of LAB application include the fermentation of cabbage into sauerkraut, the production of kimchi, and the pickling of cucumbers. Advances in biotechnology have led to the development of optimized LAB strains and starter cultures, enhancing the efficacy of preservation processes.

**Conclusion:** LAB are highly effective in preserving fruits and vegetables through multiple mechanisms, offering significant benefits in terms of extended shelf life, improved safety, enhanced nutritional value, and better sensory qualities. Ongoing research and technological advancements are expected to further improve the application of LAB in food preservation, making it a more efficient and widespread practice.

## 1. Introduction

Lactic acid bacteria (LAB) are a group of Gram-positive bacteria widely used in the food industry for their preservative properties, particularly in the fermentation of fruits and vegetables. Their role in food preservation is primarily attributed to the production of organic acids, bacteriocins, and other antimicrobial substances that inhibit the growth of spoilage organisms and pathogens [1]. The demand for natural and sustainable food preservation methods has surged in recent years, driven by consumer preferences for cleaner labels and healthier food options [2]. Recent studies have delved into various aspects of LAB's preservative effects, exploring their mechanisms of action, effectiveness in different contexts, and potential for enhancing food safety and quality [3].

For example, [4] highlighted the ability of LAB to improve the shelf life and safety of minimally processed fruits, noting significant reductions in microbial load and spoilage rates. The study emphasized the practical applications of LAB in extending the freshness of perishable produce without the need for synthetic additives. Similarly, [5, 6] investigated the impact of LAB fermentation on the nutritional profiles of vegetables. Their findings demonstrated that LAB not only preserve but also enhance the nutritional quality of vegetables by increasing the bioavailability of essential nutrients and producing beneficial metabolites. This dual benefit of preservation and nutritional enhancement positions LAB as a valuable tool in the development of functional foods.

Several authors revealed that the use of biotechnological approaches to enhance the functionality and stability of LAB strains results in more consistent and effective preservation outcomes [7]. This study is significant as it provides a comprehensive review of the recent advancements in the use of LAB for fruit and vegetable preservation. By synthesizing the latest research findings, it is aimed at offering insights into effective strategies for utilizing LAB, addressing current challenges, and highlighting the potential for future innovations in this field. The findings will be valuable for food scientists, industry professionals, and policymakers working toward safer and more sustainable food preservation methods.

### 1.1. Mechanisms of Preservation of Fruits and Vegetables Using LAB

LAB preserve fruits and vegetables through several key mechanisms. These mechanisms work synergistically to inhibit spoilage and pathogenic microorganisms, enhance nutritional value, and improve the sensory properties of the food.

#### 1.1.1. Acid Production

LAB have been of great importance in preserving fruits and vegetables, since this fermentation process results in lactic acid production, leading to a decrease in pH which makes the environment unsuitable for the growth of microorganisms and pathogens that cause spoilage. Studies show that it is also able to improve the shelf life of vegetables and fruits due to fermentation through producing lactic acid as well [8]. *Leuconostoc mesenteroides* has been used in vegetable preservation and has been reported to lower the pH, thus creating an unfavorable condition for the growth of spoilage microorganisms [9]. Furthermore, *Pediococcus pentosaceus* has been observed to produce lactic acid, which contributes to the preservation of vegetables by making the conditions acidic to the growth of microorganisms that cause spoilage [3]. Additionally, *Streptococcus thermophilus* is used in the fermentation of milk products and to preserve vegetables by its action of lactic acid formation. Similarly, another species of LAB, *Lactobacillus sakei*, is also well recognized for using a similar mechanism, that is, lactic acid production, for the protection of fruits and vegetables [10].

LAB preserve fruits and vegetables by means of homofermentation and heterofermentation, each contributing different byproducts for microbial inhibition. In the process of homofermentation, LAB produce mainly lactic acid, thereby causing a decrease in pH, hence inhibiting the growth of spoilage microorganisms and pathogens like *Listeria monocytogenes* and *Clostridium botulinum*. This pathway is very effective for preservation. During heterofermentation, LAB produce lactic acid, ethanol, and carbon dioxide, all contributing to an even more hostile environment for pathogens, with ethanol disrupting cell membranes and carbon dioxide creating anaerobic conditions. It is this activity of antimicrobial peptides precisely directed against specific pathogens that adds an extra protective layer and expands the shelf life of fermented products.

### 1.2. Bacteriocin Production

Bacteriocins are antimicrobial peptides or proteins produced by LAB that exhibit selective activity against a wide range of spoilage organisms and pathogens. Class I bacteriocins, known as lantibiotics, are small, heat-stable peptides containing unusual amino acids like lanthionine and *β*-methyllanthionine. Produced by LAB, such as *Lactococcus lactis* (nisin) and Lacticin 3147, lantibiotics act by binding to the cell membranes of target organisms, forming pores that disrupt membrane integrity and cause cell death. Nisin specifically binds to Lipid II, a key component in cell wall synthesis, inhibiting this process and further promoting bacterial death. These powerful mechanisms make lantibiotics effective inhibitors of bacterial growth, with applications in both natural and industrial settings [11].

Class II bacteriocins are small, heat-stable peptides, generally with less than 100 amino acids and without unusual amino acids; they are also called nonlantibiotics. Examples include pediocin and enterocin. The interaction of these antimicrobial bacteriocins with the bacterial membrane's lipid bilayer modifies its permeability. Intracellular components, especially ions, leak out as a result of this, which can cause cell death. Class II bacteriocins mostly exert their effects against Gram-positive bacteria, although there is a few that will work against Gram-negative bacteria as well. These properties present a number of possible applications, especially in food preservation and as substitutes for classical antibiotics [12, 13].

Class III bacteriocins are large, heat-labile proteins with a higher molecular weight than Classes I and II. These bacteriocins, such as those produced by Enterococcus species, typically target cell wall biosynthesis or inhibit enzymes inside the bacterial cell. Some also act by degrading DNA or inhibiting protein synthesis, leading to bacterial cell death [12, 14]. On the other hand, Class IV bacteriocins are complexed with lipids or carbohydrates, with notable examples produced by *Lactobacillus* species. The mode of action for Class IV bacteriocins involves intricate mechanisms, often interacting with target cell components like lipids and sugars. These interactions disrupt normal cellular functions, leading to bacterial cell damage [11, 12].

The mechanisms by which bacteriocins inhibit target microorganisms include membrane permeabilization, inhibition of cell wall synthesis, interference with DNA/RNA synthesis, and targeting specific receptors. For instance, nisin binds to Lipid II in the bacterial membrane, facilitating pore formation that disrupts membrane integrity. In summary, bacteriocins produced by LAB are vital in food preservation, especially for fruits and vegetables, due to their ability to inhibit a broad spectrum of spoilage organisms and pathogens through diverse mechanisms [15] (Table 1).

#### 1.2.1. Competition for Nutrients

LAB have the same nutritional requirements—namely, carbohydrates including glucose and fructose, amino acids, peptides, lipids, vitamins, and minerals—as the spoilage organisms and the pathogenic microorganisms. Consequently, LAB have developed specialized uptake and metabolic systems to degrade these nutrients efficiently and to outcompete other microorganisms. For example, LAB take up sugars and amino acids with the help of transport systems, making them unavailable for pathogens. Besides, LAB excrete an array of metabolic products antimicrobial in nature, namely, organic acids, hydrogen peroxide, and bacteriocins, that further put a check on the competitive microbes. Competitive exclusion mainly occurs in nutritionally poor environments, such as minimally processed fruits and vegetables that allow LAB to dominate [16–19]. This competitive exclusion is particularly effective in environments where nutrients are limited, such as minimally processed fruits and vegetables.

#### 1.2.2. Production of Other Antimicrobial Compounds

LAB produce other antimicrobial compounds, such as hydrogen peroxide and diacetyl. Hydrogen peroxide acts as an oxidizing agent that can damage microbial cells, while diacetyl has antimicrobial properties that can inhibit spoilage organisms [18, 20, 21]. These compounds further enhance the preservative effect of LAB by creating a hostile environment for undesirable microbes [22]. The detailed description of antimicrobial activities is listed in Table 2.

## 2. Enhancement of Nutritional and Sensory Properties

### 2.1. Reduction of Antinutritional Factors

LAB fermentation can increase the bioavailability of certain nutrients, such as vitamins (e.g., B vitamins) and antioxidants. These are produced by various LAB strains, with the most representative being species of *Lactobacillus*, *Bifidobacterium*, and *Pediococcus.* Phytase enzymes hydrolyze phytates into inositol and free phosphate, which effectively liberates nutritionally important minerals—like iron, calcium, and zinc—normally bound to the phytate molecule. For example, *Lactobacillus plantarum* and *Lactobacillus fermentum* have been reported to degrade phytates in fermented cereals and legumes, thereby improving the bioavailability of these essential minerals. According to [23], this enzymatic activity is one of the ways that plant-based foods, especially in regions where phytate-rich foods are a staple, can have their nutritional quality improved.

The LAB, especially the ones belonging to the genera *Lactobacillus* and *Enterococcus*, is capable of producing the enzyme tannase. It hydrolyzes tannins into less complicated compounds, which result in reduced bitterness and, therefore, make food with high tannin content more digestible. For example, L*actobacillus rhamnosus* and *Lactobacillus brevis* were found to be tannase producers during fermentation and, thus, reduce tannin content in tea and legumes, among others [24].

As a result of this enzymatic activity, the organoleptic characteristics of food, like pulses, soybeans, and tea, are enhanced, while their digestibility also improves and they become more suitable for individuals with problems due to poor nutrient absorbability because of a high tannin content. The overall flavor also improves during fermentation with LAB, where desirable aromatic compounds result. These processes emphasize the role of LAB in the enhancement of nutritional and sensory qualities of fermented [25, 26]. LAB can degrade antinutritional factors such as phytates and tannins, which bind minerals and reduce their bioavailability. The reduction of these compounds enhances the nutritional quality of the food [27]. This process is particularly beneficial in fruits and vegetables that are rich in these antinutritional factors [15]. Therefore, generally, the use of LAB in the preservation of fruits and vegetables leverages multiple mechanisms, including acid production, bacteriocin secretion, competition for nutrients, production of antimicrobial compounds, exopolysaccharides production, enhancement of nutritional and sensory properties, and reduction of antinutritional factors. These combined effects result in a comprehensive preservation strategy that extends shelf life, enhances safety, and improves the overall quality of fruits and vegetables.

### 2.2. Benefits of LAB in Fruits and Vegetable Preservation

LAB provide several significant benefits in the preservation of fruits and vegetables, offering advantages over traditional and synthetic methods. Recent research highlights these benefits, emphasizing the potential of LAB in enhancing food safety, quality, and sustainability.

#### 2.2.1. Extended Shelf Life

LAB produce lactic acid, which lowers the pH and creates an inhospitable environment for spoilage organisms. Anumudu [28] reported that LAB fermentation effectively extended the shelf life of minimally processed fruits by reducing microbial load and delaying spoilage. In addition, [16] demonstrated that fermented vegetables maintained quality and freshness for a longer duration compared to nonfermented counterparts. Traditional refrigeration also extends shelf life but requires continuous energy input and is less effective against certain spoilage organisms. Synthetic preservatives are effective but often face consumer resistance due to health concerns and regulatory restrictions.  Table 3 shows the detailed comparison of different fruits and vegetables preservation techniques (Table 3).

#### 2.2.2. Improved Safety

LAB secrete bacteriocins and other antimicrobial compounds that inhibit pathogenic bacteria. According to [15, 19], the LAB fermentation significantly reduced the presence of pathogens like *Salmonella* and *Listeria* on fresh produce. Similarly, [22] found that LAB-fermented vegetables were free from harmful bacteria throughout the storage period. Chemical preservatives can ensure safety but may pose health risks and alter the taste of food. LAB provide a natural and safer alternative without compromising the sensory qualities of the food.

The LAB have a great role in improving food safety by producing different antimicrobial metabolites, including bacteriocins, organic acids, and hydrogen peroxide, which impede the growth of pathogenic microorganisms. Bacteriocins form pores in the membranes of injurious bacteria like *Listeria monocytogenes*, *Salmonella*, and *Escherichia coli*, preventing their proliferation. In addition, LAB produces lactic acid in foods, thereby lowering pH and thus making an environment not viable for a number of pathogens. These studies carried out by [1, 30] indicated that LAB fermentation reduced harmful bacteria on fresh produce and fermented vegetables to safe storage.

While the latter have health hazards, being carcinogenic, and also affect the flavor of the food, LAB fermentation represents a more natural and safer alternative to chemical preservatives without compromise in sensory food quality. The LAB does not inhibit only the pathogenic microorganisms, but the LAB also improves the flavor of fermented foods, thereby enhancing their overall acceptability. Thus, fermentation with LAB is a natural and effective way of ensuring safety, reducing the use of chemical preservatives, and meeting the demand for healthy, more natural foods.

#### 2.2.3. Enhanced Nutritional Value

LAB increase the bioavailability of nutrients and produce beneficial metabolites during fermentation. Aguirre-Garcia [16] highlighted that LAB fermentation increased levels of B vitamins and antioxidants in vegetables, enhancing their nutritional profiles. Samtiya [27] showed that LAB fermentation reduced antinutritional factors like phytates, enhancing mineral bioavailability. Traditional methods like canning may lead to nutrient loss due to high heat processing. Synthetic additives do not enhance nutritional value and may even decrease the perceived naturalness of the product.

LAB made the nutritional value of foods significant; antinutritional factors can either be reduced or their bioavailability increased by LAB, as it increases the availability of some key nutrients. Indeed, phytates are generally abundant in cereals, legumes, and oilseeds and could further bind minerals such as iron, zinc, and calcium. LAB strains, for example, *Lactobacillus plantarum* and *Bifidobacterium*, bear phytase enzymes that depolymerize phytates into inositol and free phosphate, thereby releasing the bound minerals and hence improving their bioavailability. Other polyphenolic compounds like tannins, present in some of the foods, inhibit protein and mineral absorption; LAB strains like *Lactobacillus rhamnosus* and *Lactobacillus brevis*, on the other hand, produce during fermentation tannase enzymes that reduce bitterness, hence improving digestibility. LAB fermentation also eliminates the protease inhibitors present in legumes, seeds, and grains, which impede protein digestion. Some strains have been reported to degrade these inhibitors, improving protein bioavailability. Besides that, fermentation by LAB generally increases the bioavailability of B vitamins—especially folate—and important minerals such as iron, calcium, and magnesium due to its degradation of phytates or other substances impeding nutrient absorption. LAB also enhance the bioavailability of amino acids, degrading complex proteins into peptides and free amino acids, which generally improve the digestibility and absorption of proteins, but especially in fermented pulses. All these processes contribute to an improved nutritional profile of fermented foods, highly beneficial for human consumption (Table 4).

#### 2.2.4. Improved Sensory Attributes

LAB produce organic acids, exopolysaccharides, and other compounds that enhance flavor, aroma, and texture. Katepogu et al. [31, 32] reported that LAB-fermented fruits and vegetables had better taste, aroma, and texture compared to nonfermented ones. [33] found that exopolysaccharide production by LAB improved the mouthfeel and overall sensory appeal of fermented products. Synthetic flavor enhancers can improve sensory attributes but may be perceived negatively by consumers seeking natural products. LAB offer a natural enhancement of sensory qualities without the need for artificial additives.

## 3. Environmental and Economic Benefits

The preservation of vegetables and fruits is done to prevent losses and ensure the security of food throughout the year by making them available. This is generally done through refrigeration, freezing, drying, canning, and fermentation, which have different environmental and economic implications. Refrigeration and freezing are the most used methods that preserve freshness; through these, microbial activity can be retarded. However, all these techniques are highly energy-intensive, requiring steady electricity to maintain low temperatures, thus contributing highly to greenhouse gas emission [34]. Economically, refrigeration and freezing involve high operational costs regarding energy and equipment maintenance, thus making the technologies not very accessible to small-scale producers or areas where there is limited electricity.

Drying and dehydration techniques rely on moisture removal as the critical factor inhibiting the growth of microorganisms. Traditional drying, such as sun drying, is environmentally friendly but depends on weather conditions. On the other hand, industrial dehydration generally requires huge energy inputs, mostly for hot air or vacuum drying methods [35]. These methods greatly extend shelf life and reduce transportation costs because of lower weight, but high initial investment in equipment is a barrier to entry for small businesses. Canning also employs the method of heat treatment and airtight sealing, giving the produce a long shelf life and safety from microbes. The process is, however, energy intensive, dependent on nonbiodegradable packaging material, and consequently leads to significant generation of waste; hence, it is considered an environmentally concerning method [33].

Chemical preservation is generally based on adding synthetic or natural preservatives to inhibit the growth of microorganisms to prevent spoilage. Although the methods are usually inexpensive and technically easy to conduct, their uses are quite general, but sometimes, chemical production and disposal lead to chemical wastes and environmental pollution. Besides, consumer demand has now changed to clean-label products over residues of chemicals, making the quest for their alternatives quite important [30].

## 4. Application Strategy of LAB as Preservatives on Fruits and Vegetables

LAB provide an effective and natural means of preserving various fruits and vegetables, including bananas, oranges, avocados, papayas, carrots, tomatoes, and potatoes. Recent research has highlighted specific strategies tailored to each type of produce to maximize the benefits of LAB preservation.

For bananas, LAB can be applied as a protective coating or dip to extend shelf life and reduce spoilage. Suriati [36] demonstrated that a LAB coating significantly delayed ripening and reduced microbial spoilage, maintaining the quality of bananas during storage. This process involves applying a solution of LAB strains to the surface of the bananas, which acts as a barrier against oxygen and moisture, slowing down the ripening process and inhibiting the growth of spoilage microorganisms.

In the case of oranges, LAB can be used in the form of a wash or dip to reduce microbial load and extend shelf life. Yan et al. [37] found that LAB treatments on oranges reduced postharvest decay caused by fungal pathogens and maintained fruit quality during storage. The LAB wash can be incorporated into the packing line, where oranges are briefly soaked or sprayed with the LAB solution. This treatment not only reduces the initial microbial load but also establishes a beneficial microbial community on the fruit's surface, which can outcompete and suppress pathogenic microbes.

For avocados, LAB fermentation can be used to produce avocado-based fermented products, enhancing shelf life and nutritional value. Hence, LAB fermentation of avocado pulp improved its antioxidant properties and extended the product's shelf life by inhibiting microbial growth [38]. Avocado pulp can be inoculated with specific LAB strains and allowed to ferment under controlled conditions. This process increases the production of beneficial compounds such as vitamins, antioxidants, and antimicrobial peptides, which contribute to both extended shelf life and improved health benefits.

Similarly, for papayas, LAB can be applied as a coating or included in packaging to prevent spoilage and maintain quality. Garofalo et al. [38] showed that LAB coatings on papayas significantly delayed ripening and reduced the incidence of fungal spoilage, thereby extending the fruit's shelf life. The LAB coating forms a protective film on the surface of the papayas, reducing the respiration rate and ethylene production, which are key factors in the ripening process. Additionally, the antimicrobial compounds produced by LAB inhibit the growth of spoilage fungi.

In the realm of vegetables, carrots can benefit from LAB fermentation, producing slices or juice with enhanced shelf life and nutritional value. Ebah et al. [39] found that LAB fermentation of carrot slices resulted in a product with prolonged shelf life, improved safety, and enhanced levels of bioactive compounds. Carrot slices can be inoculated with LAB and stored in anaerobic conditions to allow fermentation. This not only preserves the texture and color of the carrots but also increases their probiotic content, providing additional health benefits to consumers.

For tomatoes, LAB can be applied as a postharvest treatment to prevent spoilage and maintain quality. Ungureanu et al. [40] demonstrated that LAB treatments on tomatoes reduced spoilage and maintained firmness and color during storage. Tomatoes can be dipped or sprayed with a LAB solution after harvesting. This treatment helps in maintaining the structural integrity of the tomatoes by preventing the breakdown of cell walls, which is often caused by spoilage organisms. The LAB treatment also helps in maintaining the natural flavor and aroma of the tomatoes by preventing off-flavors typically associated with spoilage.

Lastly, for potatoes, LAB can be used in the form of a dip or spray to prevent spoilage and extend shelf life. Khorramifar et al. [41] showed that LAB treatments on potatoes inhibited the growth of spoilage organisms and maintained tuber quality during storage. Potatoes can be treated with a LAB solution either before storage or during packing. This treatment forms a protective layer on the surface of the potatoes, reducing the risk of bacterial and fungal infections. The LAB also produces enzymes that can break down toxins produced by spoilage organisms, ensuring the safety and quality of the potatoes.

Overall, the application of LAB as preservatives for fruits and vegetables has shown promising results in extending shelf life, enhancing nutritional value, and maintaining quality. Different strategies, including coatings, dips, washes, and fermentation, have been effectively employed across various types of produce. These methods offer natural and sustainable alternatives to traditional and synthetic preservation techniques. By leveraging the natural antimicrobial properties and beneficial effects of LAB, it is possible to reduce reliance on chemical preservatives and minimize the environmental impact of food preservation (Table 5).

Further research and technological innovations in the application of LAB will keep on improving their applicability in food preservation. Innovations in optimized strain selection and genetic engineering will enhance the antimicrobial properties of LAB, hence the fight against spoilage organisms. Innovations related to controlled fermentation conditions, along with advances in packaging technologies, will make LAB preservation more economical and industrially feasible with the advent of biodegradable or edible LAB-infused packaging. Combining LAB with other methods of natural preservation will further improve the quality and safety of foods. These will add strength to LAB regarding sustainable food preservation without food wastes and dependence on chemical preservatives.

Other research is currently ongoing, with technology improving in the use of LAB in enhanced means of food preservation; for example, in bananas, using *Lactobacillus plantarum* in a protective coat prolongs their freshness and delays fruit ripening for 7–14 days at least, hence delaying microbial degradation [45]. Oranges treated with *Lactobacillus rhamnosus* showed an extension of shelf life to 10 days by reducing postharvest decay, maintaining the keeping quality of fruits [46].

Similarly, LAB-fermented avocado pulp with *Lactobacillus acidophilus* extends its shelf life from 10 to 15 days. It enhances the nutritional values and antioxidants present therein [38]. While the papaya samples treated with coatings made with *Lactobacillus casei* retain quality during all 12 days of storage, they maintain a great delay from both physiological spoilages [38]. On the other hand, LAB-fermented carrots—if fermented, taking the time allowance to ferment the raw vegetables with *Lactobacillus brevis* can—increase their shelf life from 14 to 21 days by enhancing safety with improvements in the probiotic content [47].

## 5. Recommendation

Based on the research outcomes, the following recommendations can be made for the application of LAB as preservatives on fruits and vegetables:

Further research should focus on optimizing LAB application methods, such as coatings, dips, washes, and fermentation, to maximize their preservative effects while minimizing resource use. Investigate the strain-specific effects of LAB on different types of produce to identify the most effective strains for specific preservation goals. Explore integration with innovative packaging technologies, such as active and intelligent packaging, to enhance preservation efficacy and prolong shelf life. Evaluate the synergistic effects of combining LAB with other preservation techniques, such as modified atmosphere packaging or natural antimicrobial compounds, to further enhance preservation outcomes.

### 5.1. Future Perspectives

The future of LAB as preservatives for fruits and vegetables holds promising opportunities and challenges: Explore the use of LAB consortia and microbiome-based approaches to leverage synergistic interactions among beneficial microorganisms for enhanced preservation effects. Harness biotechnological advancements, such as genetic engineering and probiotic formulations, to develop customized LAB strains with tailored preservation capabilities. Address consumer perceptions and regulatory aspects related to natural preservatives, ensuring transparency, safety, and compliance with food standards. Integrate LAB-based preservation strategies into broader sustainable food systems initiatives, emphasizing reduced food waste, resource efficiency, and environmental conservation.

## Figures and Tables

**Table 1:** Bacteriocins and their properties.

**Bacteriocin**	**Class**	**Target organisms**	**Mode of action**	**Example strains**
Nisin	Class I (lantibiotic)	*Listeria monocytogenes*, *Clostridium botulinum*	Membrane pore formation, inhibition of cell wall biosynthesis	*Lactococcus lactis*
Pediocin	Class II (nonlantibiotic)	*Listeria monocytogenes*	Membrane disruption, leakage of cell contents	*Pediococcus acidilactici*
Enterocin	Class II (nonlantibiotic)	*Enterococcus faecalis*, *Enterococcus faecium*	Membrane disruption, cell leakage	*Enterococcus faecium*
Lacticin 3147	Class I (lantibiotic)	*Listeria monocytogenes*, *Staphylococcus aureus*	Membrane disruption	*Lactococcus lactis*

**Table 2:** Antimicrobial compounds, sources of organisms, and their mode of action.

**Category**	**Antimicrobial compound**	**Source organism**	**Mode of action**	**Examples**
Bacteriocins	Lantibiotics (Class I)	Lactic acid bacteria (e.g., *Lactococcus lactis*)	Disrupts cell membrane, inhibits cell wall synthesis	Nisin, Lacticin 3147
	Nonlantibiotics (Class II)	Lactic acid bacteria (e.g., *Pediococcus* species)	Alters membrane permeability, causing leakage of cellular contents	Pediocin, enterocin
	Large bacteriocins (Class III)	*Enterococcus* species	Targets cell wall biosynthesis, inhibits enzymes, degrades DNA	Enterocin A, bacteriocins produced by *Enterococcus*

Antibiotics	Penicillin	*Penicillium* species	Inhibits cell wall synthesis by targeting peptidoglycan biosynthesis	Penicillin
	Streptomycin	*Streptomyces griseus*	Inhibits protein synthesis by binding to bacterial ribosomes	Streptomycin
	Tetracycline	*Streptomyces aureofaciens*	Inhibits protein synthesis by binding to the bacterial ribosome	Tetracycline

Fungal metabolites	Griseofulvin	*Penicillium griseofulvum*	Disrupts fungal cell division by binding to microtubules	Griseofulvin
	Echinocandins	*Glarea lozoyensis*	Inhibits synthesis of glucan in fungal cell walls	Caspofungin, micafungin
	Aflatoxins	*Aspergillus* species	Inhibits protein synthesis, causes DNA damage	Aflatoxin B1, Aflatoxin B2

Plant secondary metabolites	Alkaloids	Various plant species (e.g., *Capsicum* species)	Inhibits microbial growth by disrupting cell processes	Capsaicin, berberine
	Terpenoids	Various plants (e.g., *Thymus vulgaris*)	Disrupts microbial membrane integrity	Thymol, carvacrol

Animal-derived compounds	Defensins	Mammals (e.g., humans, amphibians)	Disrupts bacterial membrane integrity	Human defensin, anuran defensins
	Lactoferrin	Mammals (e.g., cows and humans)	Binds iron, depriving bacteria of this essential nutrient	Lactoferrin

Polypeptides	Bacitracin	*Bacillus subtilis*	Interferes with bacterial cell wall synthesis	Bacitracin

Other microbial compounds	Hydrogen peroxide	Various microorganisms (e.g., *Lactobacillus* species)	Oxidizes and damages cellular components like lipids and proteins	Hydrogen peroxide
	Organic acids	Lactic acid bacteria, *Saccharomyces* species	Lowers pH, inhibits bacterial growth	Lactic acid, acetic acid

**Table 3:** Comparison of methods for extending shelf life of fruits and vegetables.

**Method**	**Description**	**Advantages**	**Disadvantages**
Refrigeration/controlled atmosphere	Storage at low temperature or altered atmosphere to slow ripening and microbial growth	Preserves texture, flavor, and nutritional value	Limited shelf life, high energy cost for refrigeration
Freezing	Freezing slows microbial growth and enzyme activity	Long shelf life, preserves nutrients and taste	Texture changes in some fruits and vegetables, requires storage space
Canning	High heat applied to destroy microorganisms and enzymes, sealing in containers	Long shelf life, convenient for long-term storage	Loss of texture and some nutrients, high energy consumption
Drying	Removal of moisture using heat or air, inhibiting microbial growth	Low cost, retains flavors, easy to store	Loss of texture, can be labor-intensive, may alter flavor
Fermentation	Microbial conversion of sugars into acids, creating an acidic environment	Enhances flavor, improves digestibility, extends shelf life	Requires time and controlled conditions, may not be suitable for all vegetables
Vacuum packaging	Removal of air and sealing food in vacuum-sealed bags	Extends shelf life by reducing oxygen, preserves freshness	Requires packaging equipment, limited to refrigerated or frozen storage
Coating/edible films	Natural films or coatings reduce moisture loss and oxidation	Natural method, retains moisture and prevents spoilage	May alter texture, not suitable for all fruits/vegetables

*Note: Source:* [29].

**Table 4:** Strategies for reducing antinutritional factors and improving bioavailability.

**Nutrient/antinutrient**	**Antinutritional factor**	**Method to reduce antinutrient**	**Bioavailability enhancement strategy**
Iron	Phytates, tannins, calcium	Soaking, sprouting, fermentation, heat processing	Vitamin C-rich foods, fermentation, reduce inhibitors (tannins, calcium)
Zinc	Phytates	Soaking, sprouting, fermentation	Phytate reduction, animal protein, citric acid
Calcium	Oxalates	Decortication, fermentation, alkaline processing	Avoid high oxalate foods, fermentation, alkaline processing
Vitamin A (beta-carotene)	None (conversion to active form)	None	Fat inclusion, fermentation
Folate	None (heat and light sensitivity)	Light cooking, steaming	Fermentation, light cooking/steaming

**Table 5:** LAB preservation strategies for fruits and vegetables and their effectiveness.

**Fruit/vegetable**	**LAB preservation strategy**	**Effectiveness and outcome**	**References**
Bananas	LAB coating or dip	Delays ripening, reduces microbial spoilage, extends shelf life	Suriati [36]
Oranges	LAB wash or dip	Reduces fungal decay, maintains quality, extends shelf life	Yan et al. [42]
Avocados	LAB fermentation of pulp	Enhances antioxidant properties, extends shelf life, improves nutritional value	Garofalo et al. [38]
Papayas	LAB coating	Delays ripening, reduces fungal spoilage, maintains quality	Garofalo et al. [38]
Carrots	LAB fermentation of slices or juice	Prolongs shelf life, enhances safety, increases probiotic content and bioactive compounds	Ebah et al. [43]
Tomatoes	LAB postharvest treatment	Prevents spoilage, maintains firmness, color, and natural flavor	Ungureanu et al. [44]
Potatoes	LAB dip or spray	Inhibits microbial growth, reduces spoilage, maintains tuber quality	Khorramifar et al. [41]

## Data Availability

No additional supporting data is available, and all data was confidentially used in the main document.

## References

[1] Zapaśnik A., Sokołowska B., Bryła M. (2022). Role of Lactic Acid Bacteria in Food Preservation and Safety. *Foods*.

[2] Bürck M., Ramos S. . P., Braga A. R. C. (2024). Enhancing the Biological Effects of Bioactive Compounds from Microalgae Through Advanced Processing Techniques: Pioneering Ingredients for Next-Generation Food Production. *Foods*.

[3] Rathod N. B., Phadke G. G., Tabanelli G. (2021). Recent Advances in Bio-Preservatives Impacts of Lactic Acid Bacteria and Their Metabolites on Aquatic Food Products. *Food Bioscience*.

[4] De Corato U. (2020). Improving the Shelf-Life and Quality of Fresh and Minimally-Processed Fruits and Vegetables for a Modern Food Industry: A Comprehensive Critical Review From the Traditional Technologies Into the Most Promising Advancements. *Critical Reviews in Food Science and Nutrition*.

[5] Knez E., Kadac-Czapska K., Grembecka M. (2023). Effect of Fermentation on the Nutritional Quality of the Selected Vegetables and Legumes and Their Health Effects. *Life*.

[6] Naseem A., Akhtar S., Ismail T. (2023). Effect of Growth Stages and Lactic Acid Fermentation on Anti-Nutrients and Nutritional Attributes of Spinach (*Spinacia Oleracea*). *Microorganisms*.

[7] Rachwał K., Gustaw K. (2024). Lactic Acid Bacteria in Sustainable Food Production. *Sustainability*.

[8] Di Cagno R., Coda R., De Angelis M., Gobbetti M. (2013). Exploitation of Vegetables and Fruits Through Lactic Acid Fermentation. *Food Microbiology*.

[9] Fleming H. P., McFeeters R. F., Daeschel M. A. (2018). The Lactobacilli, Pediococci, and Leuconostocs: Vegetable Products. *Bacterial Starter Cultures for Food*.

[10] Tamang J. P., Shin D. H., Jung S. J., Chae S. W. (2016). Functional Properties of Microorganisms in Fermented Foods. *Frontiers in Microbiology*.

[11] Simons A., Alhanout K., Duval R. E. (2020). Bacteriocins, Antimicrobial Peptides from Bacterial Origin: Overview of Their Biology and Their Impact Against Multidrug-Resistant Bacteria. *Microorganisms*.

[12] Darbandi A., Asadi A., Ghanavati R. (2021). The Effect of Probiotics on Respiratory Tract Infection With Special Emphasis on COVID-19: Systemic Review 2010–20. *International Journal of Infectious Diseases*.

[13] Yu K., Huang Z., Li Y. (2021). Establishment and Application of Matrix-Assisted Laser Desorption/Ionization Time-of-Flight Mass Spectrometry for Detection of *Shewanella* Genus. *Frontiers in Microbiology*.

[14] Linares-Morales J. R., Gutiérrez-Méndez N., Rivera-Chavira B. E., Pérez-Vega S. B., Nevárez-Moorillón G. V. (2018). Biocontrol Processes in Fruits and Fresh Produce, the Use of Lactic Acid Bacteria as a Sustainable Option. *Frontiers in Sustainable Food Systems*.

[15] Mokoena M. P., Mutanda T., Olaniran A. O. (2016). Perspectives on the Probiotic Potential of Lactic Acid Bacteria From African Traditional Fermented Foods and Beverages. *Food & Nutrition Research*.

[16] Aguirre-Garcia Y., Nery-Flores S. D., Campos-Muzquiz L. G. (2024). Lactic Acid Fermentation in the Food Industry and Bio-Preservation of Food. *Fermentation*.

[17] Amenu D., Desalegn R., Nugusa A., Tolera C., Tafesse T. (2024). Evaluation of Antifungal Activity of Plant Extracts Against Plant Pathogen Associated With Sweet Orange (*Citrus sinensis* L.), in Jimma, Western Ethiopia. *Discover Applied Sciences*.

[18] Amenu D., Bacha K. (2025). Antagonistic Effects of Lactic Acid Bacteria Isolated From Ethiopian Traditional Fermented Foods and Beverages Against Foodborne Pathogens. *Probiotics and Antimicrobial Proteins*.

[19] Cirat R., Capozzi V., Benmechernene Z., Spano G., Grieco F., Fragasso M. (2024). LAB Antagonistic Activities and Their Significance in Food Biotechnology: Molecular Mechanisms, Food Targets, and Other Related Traits of Interest. *Fermentation*.

[20] Amenu D., Bacha K. (2023). Probiotic Potential and Safety Analysis of Lactic Acid Bacteria Isolated From Ethiopian Traditional Fermented Foods and Beverages. *Annals of Microbiology*.

[21] Ammor S., Tauveron G., Dufour E., Chevallier I. (2006). Antibacterial Activity of Lactic Acid Bacteria Against Spoilage and Pathogenic Bacteria Isolated From the Same Meat Small-Scale Facility: 1—Screening and Characterization of the Antibacterial Compounds. *Food Control*.

[22] Verma D. K., Thakur M., Singh S. (2022). Bacteriocins as Antimicrobial and Preservative Agents in Food: Biosynthesis, Separation and Application. *Food Bioscience*.

[23] Haug W., Lantzsch H. J. (1983). Sensitive Method for the Rapid Determination of Phytate in Cereals and Cereal Products. *Journal of the Science of Food and Agriculture*.

[24] Darbandi A., Asadi A., Mahdizade Ari M. (2022). Bacteriocins: Properties and Potential Use as Antimicrobials. *Journal of Clinical Laboratory Analysis*.

[25] Behera S. S., Ray R. C., Zdolec N. (2018). Lactobacillus plantarum With Functional Properties: An Approach to Increase Safety and Shelf-Life of Fermented Foods. *BioMed Research International*.

[26] Rollán G. C., Gerez C. L., Leblanc J. G. (2019). Lactic Fermentation as a Strategy to Improve the Nutritional and Functional Values of Pseudocereals. *Frontiers in Nutrition*.

[27] Samtiya M., Aluko R. E., Dhewa T. (2020). Plant Food Anti-Nutritional Factors and Their Reduction Strategies: An Overview. *Food Production, Processing and Nutrition*.

[28] Anumudu C., Hart A., Miri T., Onyeaka H. (2021). Recent Advances in the Application of the Antimicrobial Peptide Nisin in the Inactivation of Spore-Forming Bacteria in Foods. *Molecules*.

[29] Nikolić N., Mitrović J., Karabegović I. (2019). A Comparison Between Wheat and Different Kinds of Corn Flour Based on Minerals, Free Phenolic Acid Composition and Antioxidant Activity. *Quality Assurance and Safety of Crops & Foods*.

[30] Ibrahim S. A., Ayivi R. D., Zimmerman T. (2021). Lactic Acid Bacteria as Antimicrobial Agents: Food Safety and Microbial Food Spoilage Prevention. *Foods*.

[31] Katepogu H., Wee Y. J., Almaary K. S. (2022). Isolation and Characterization of *Pediococcus* sp. HLV1 From Fermented Idly Batter. *Fermentation*.

[32] Bao Y., Zhang Y., Li H. (2012). In Vitro Screen of *Lactobacillus plantarum* as Probiotic Bacteria and Their Fermented Characteristics in Soymilk. *Annals of Microbiology*.

[33] Islam M., Xayachak T., Haque N., Lau D., Bhuiyan M., Pramanik B. K. (2024). Impact of Bioplastics on Environment From Its Production to End-of-Life. *Process Safety and Environment Protection*.

[34] Park W. Y., Shah N., Phadke A. (2019). Enabling Access to Household Refrigeration Services Through Cost Reductions From Energy Efficiency Improvements. *Energy Efficiency*.

[35] Skendi A., Bouloumpasi E., Chatzopoulou P., Biliaderis C. C., Irakli M. (2023). Comparison of Drying Methods for the Retention of Phenolic Antioxidants in Post-Distillation Solid Residues of Aromatic Plants. *LWT*.

[36] Suriati L. (2022). Nano Coating of Aloe-Gel Incorporation Additives to Maintain the Quality of Freshly Cut Fruits. *Frontiers in Sustainable Food Systems*.

[37] Yan X., Dong T., Yun X., Liu B., Meng J. (2022). Enhancing the Functionality of Cross-Linked Chitosan Coating on Vibration Damaged Nanguo Pears. *LWT*.

[38] Garofalo G., Ponte M., Busetta G. (2024). Microbial Dynamics and Quality Characteristics of Spontaneously Fermented Salamis Produced by Replacing Pork Fat With Avocado Pulp. *Food Microbiology*.

[39] Ebah E. E., Wusuum B., Akande T., Emmanuel O. O., Ikala R. O., Ode T. A. (2019). Effect of Lactic-Acid Fermentation on the Shelf Life of Vegetables. *The American Journal of Innovative Research & Applied Sciences*.

[40] Ungureanu C., Tihan G., Zgârian R., Pandelea (Voicu) G. (2023). Bio-Coatings for Preservation of Fresh Fruits and Vegetables. *Coatings*.

[41] Khorramifar A., Rasekh M., Karami H. (2023). Determining the Shelf Life and Quality Changes of Potatoes (*Solanum tuberosum*) During Storage Using Electronic Nose and Machine Learning. *PLoS One*.

[42] Yan P. D., Oulahal N. (2025). Bioprotective Yeasts: Potential to Limit Postharvest Spoilage and to Extend Shelf Life or Improve Microbial Safety of Processed Foods. *Heliyon*.

[43] Ebah E. E., Odo J. I., Dickson I. O. E., Iqbal M. N. (2024). Biodegradation of Combine Tributyltin and Diphenyltin by Bacteria in Freshwater Sediment. *International Journal of Molecular Microbiology*.

[44] Ungureanu G., Enache I. M., Cara I. G., Motrescu I., Patras A. (2024). Insights Into the Environmental Benefits of Using Apple Pomace for Biosorption of Lead From Contaminated Water. *Heliyon*.

[45] Islam S., Biswas S., Jabin T. (2023). Probiotic Potential of *Lactobacillus plantarum* DMR14 for Preserving and Extending Shelf Life of Fruits and Fruit Juice. *Heliyon*.

[46] Romero J., Albertos I., Díez-Méndez A., Poveda J. (2022). Control of Postharvest Diseases in Berries Through Edible Coatings and Bacterial Probiotics. *Scientia Horticulturae*.

[47] Nwoko M. C., Achufusi J. N., Ahaiwe M. C., Ehumadu C. R. (2018). Evaluation of Yield, Heavy Metals and Vitamins Compositions of *Pleurotus pulmonarius* (Fries) Quell. Fruit Bodies Cultivated on Three Deciduous Tree Logs. *Journal of Environmental Science and Public Health*.

